# Application of real time shear wave elastography to the differential diagnosis of secondary parathyroid hyperplasia and adenoma

**DOI:** 10.1097/MD.0000000000035079

**Published:** 2023-09-15

**Authors:** Xiang-Yi Li, Hao-Wen Li, Yong-Kang Liu

**Affiliations:** a China Aerospace Science and Industry Corporation 731 Hospitaloptional, Feng Tai District, Beijing, China.

**Keywords:** differential diagnosis, medical imaging, parathyroid adenoma, parathyroid hyperplasia, real time shear wave elastography, ultrasonography

## Abstract

We aimed to explore the value of ultrasonic elastic imaging in the diagnosis of parathyroid hyperplasia and adenoma in patients with secondary hyperparathyroidism and provide more evidence for clinical treatment. Forty patients who were on dialysis and underwent parathyroid surgery were selected All patients underwent routine ultrasound, ultrasound elasticity examination and blood biochemical examination before surgery, including calcium, phosphorus, parathyroid hormone (PTH), etc. According to postoperative results, adenoma group and hyperplasia group were divided into 2 groups. Receiver operating characteristic curve was drawn to evaluate the diagnostic efficacy and combined diagnostic efficacy of each index. The PTH levels significantly differed between the adenoma and hyperplasia groups (*P* < .001). The volume and blood flow grades significantly differed between the adenoma and hyperplasia groups (*P* < .001) The minimum of the adenoma group was 14.62 ± 6.79 kPa, mean was 19.42 ± 6.29 kPa, and maximum was 24.25 ± 6.35 kPa which were significantly different from those in the hyperplasia group (*P* < .05). The combinations of more than 6 indicators in the diagnosis of parathyroid adenoma resulted in an area under the curve of 0.892 (*P* < .001), and the sensitivity and specificity were 78.9% and 97.4%, respectively. Shear wave elastography can be used as an effective tool to distinguish secondary parathyroid hyperplasia from adenoma. When combined with PTH, conventional ultrasound blood flow grading and volume measurement, it has higher diagnostic efficacy.

## 1. Introduction

Disorders of calcium, phosphorus, and vitamin D metabolism in patients with chronic renal insufficiency stimulate excessive secretion of parathyroid hormone (PTH), leading to secondary hyperparathyroidism (sHPT), which progresses to hyperplasia and adenoma, the most common complications of end-stage renal disease. An excessive imbalance in calcium and phosphorus levels in patients with sHPT causes calcium deposition in multiple organs, vascular calcification, osteoporosis, and pathological fractures, which increases the disability and fatality rates of patients with kidney disease and significantly reduces their quality of life.^[1–3]^ Both parathyroid adenomas and parathyroid hyperplasia are abnormalities of normal parathyroid tissue caused by excessive parathyroid hormone secretion. However, the symptoms of the 2 are different and the treatment methods are different, so it is necessary to distinguish between them during treatment. Parathyroid adenomas are caused by a benign tumor of one of the parathyroid glands, and hyperplasia involves enlargement of all 4 parathyroid glands. Surgical removal of the affected gland cures parathyroid adenoma, whereas hyperplasia requires removal of 3 semiglands to control the condition. Currently, surgery is the preferred treatment for parathyroid hyperplasia and adenoma that cannot be controlled by drugs. The accurate identification of lesions before surgery can aid in the formulation of surgical protocols.

Ultrasonography is safe, noninvasive, and reproducible and is the first choice for examining the parathyroid glands. Ultrasonic elastography can distinguish tissue hardness and provide qualitative and quantitative information, known as “acoustic palpation imaging,” which is a vital supplement to traditional ultrasound. Currently, it is widely used in the examination of the liver, thyroid, breast, and other organs, and ultrasonic elastic imaging of the parathyroid gland has recently been explored.^[4–6]^ However, we require further evidence supporting the use of ultrasonic elastography in the diagnosis of parathyroid hyperplasia and adenoma in patients with sHPT. Further investigation must be conducted to determine the accuracy and reliability of ultrasonic elastography in identifying parathyroid lesions.

Therefore, we aimed to explore the value of ultrasonic elastic imaging in the diagnosis of parathyroid hyperplasia and adenoma in patients with sHPT and provide more evidence for clinical treatment.

## 2. Materials and methods

### 2.1. General information

Forty patients who were on dialysis and underwent parathyroid surgery between 2019 and 2022 were selected in China Aerospace Science and Industry Corporation 731 Hospital. The inclusion criteria were as follows: Patients who underwent parathyroid surgery and were diagnosed with parathyroid adenoma or parathyroid hyperplasia by histopathology; Conventional high-frequency ultrasound and shear wave elastography (SWE) were performed before surgery; and Patients who underwent blood biochemical examination, including calcium, phosphorus, PTH concentrations, and had corrected values calculated from the raw measurement values. Exclusion criteria: patients requiring emergency surgery, complicated with severe cardiovascular disease, cerebrovascular disease, pulmonary disease, and abnormal liver and kidney function. Consequently, the cohort consisted of 22 men and 18 women, aged 39 to 92 years, with an average age of 63.9 ± 14.1 years. A total of 57 specimens, including 19 adenomas and 38 hyperplasia samples, were obtained from these patients after surgery. Dialysis was performed 3 times a week for 5 to 18 years. Sex- or age-related differences were not significant between the 2 groups (*P* > .05). All patients signed an informed consent form, and the study was approved by the Ethics Committee of our hospital.

### 2.2. Instruments and methods

#### 2.2.1. Routine ultrasound examination.

A superficial ultrasonic probe with frequencies of 12 to 5 MHz was selected using a Mindray resona7 color Doppler ultrasound Instrument-model. The patient was instructed to lie on their back, remove any neck accessories, and fully expose the anterior neck area. Scanning was performed from the mandibular angle to the sternum and supraclavicular fossa. After the parathyroid glands were scanned, their location, number, size, shape, internal echo, and blood supply of the parathyroid glands were comprehensively recorded. The volume of the parathyroid gland was calculated according to the formula: volume (cm^3^) = 1/6π × length × width × height. According to the Adler semiquantitative method, the blood flow signals of the glands were graded as follows: level 0, no blood flow signal, and level 1, minimal peripheral or central blood flow signal. Level 2 occurs when the blood flow signal is > 30% of the circumference of the gland or < 30% of its surface. Level 3 occurs when peripheral and central blood flow signals are both 30% greater than those of the glandular surface.

#### 2.2.2. Ultrasonic elastography.

**Figure 1.**
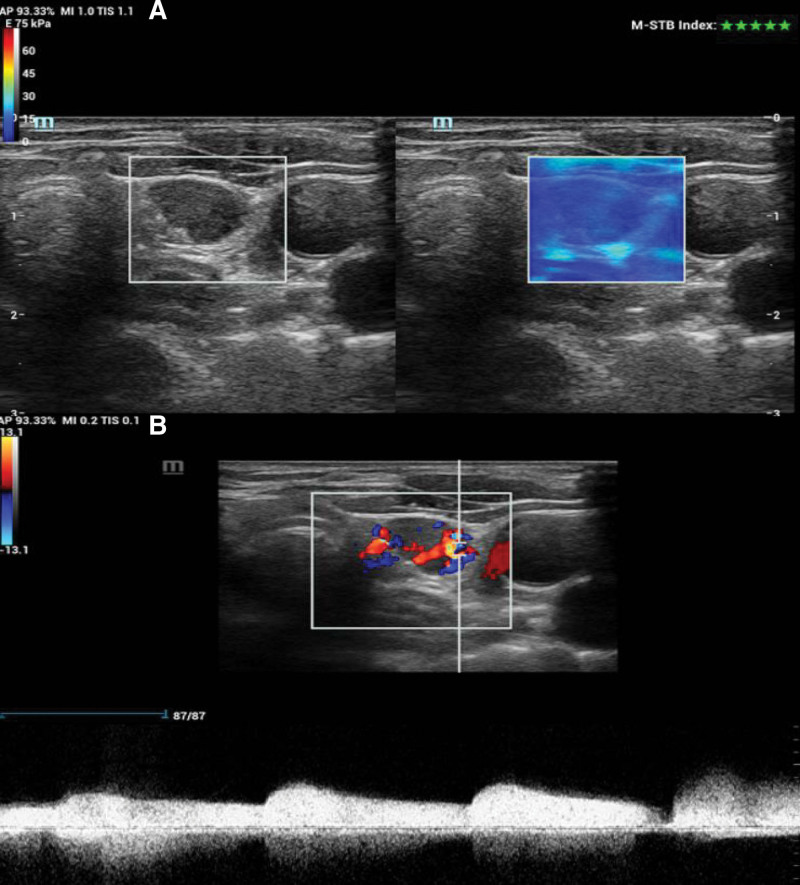
(A) E-max, E-mean, and E-min of the parathyroid adenoma were 17.61 kPa, 16.29 kPa, and 15.00 kPa, respectively. (B) The lesion exhibits grade 3 blood flow. E-min = minimum, E-max = maximum.

**Figure 2.**
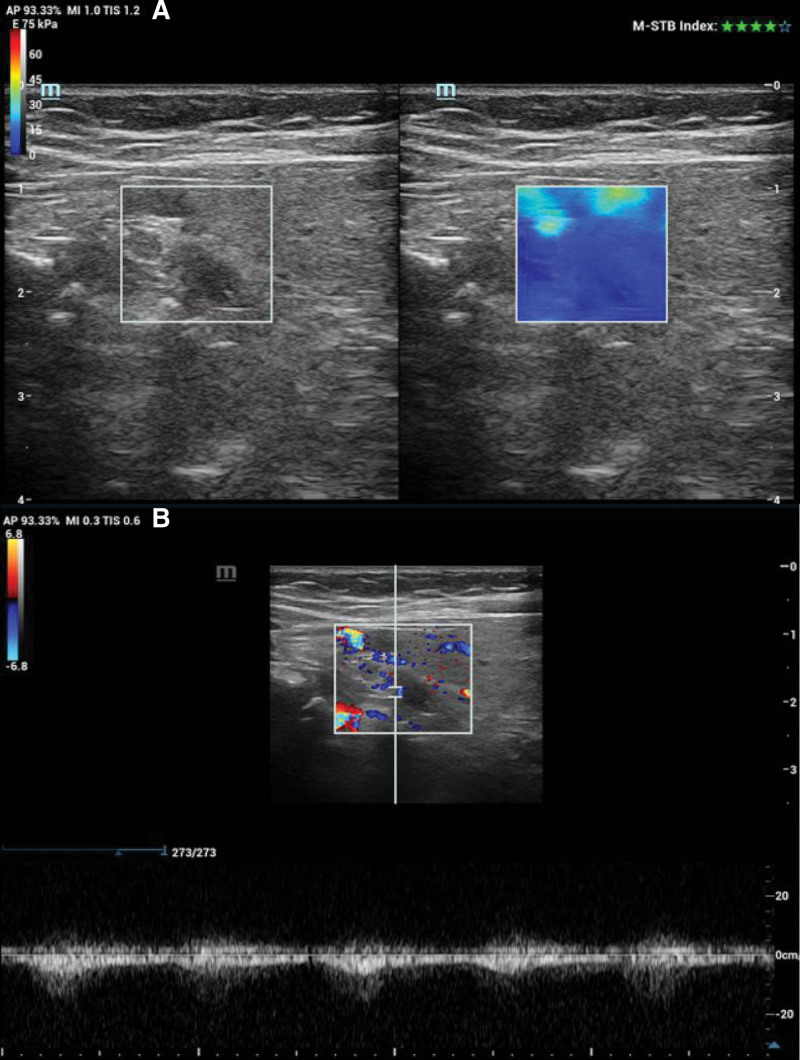
(A) E-max, E-mean, and E-min of the parathyroid hyperplasia glands were 8.55 kPa, 7.43 kPa, and 6.74 kPa, respectively. (B) The lesion exhibits grade 1 blood flow. E-min = minimum, E-max = maximum.

By applying a sufficient coupling agent, the probe was gently placed on the surface of the body, and the SWE mode was enabled on the longitudinal section. Next, the target parathyroid gland was placed in the region of interest, part of the thyroid and surrounding tissue was covered, the patient was asked to hold their breath, the image was evenly filled, incubated for 2 to 3 seconds, then frozen and stored. The mean (E-mean), minimum (E-min), and maximum (E-max) values of the elastic modulus of the parathyroid gland were measured, and these steps were repeated 5 times to obtain the mean value for evaluating the elasticity of the parathyroid gland (Figs. 1 and 2).

### 2.3. Statistical analysis

The SPSS 22 software was used for the statistical analysis. Normally distributed measurement data are expressed as mean ± standard deviation (x ± s). The t-test was used to compare independent samples, and the chi-square test was used to compare count data, with the test level α = 0.05. Receiver operating characteristic curves were constructed, and the area under the curve (AUC) was calculated to determine the optimal diagnostic threshold.

## 3. Results

### 3.1. Comparison of the preoperative blood biochemical results of patients

The PTH levels significantly differed between the adenoma and hyperplasia groups (*P* < .001), but no statistically significant difference in blood calcium and phosphorus levels (*P* > .05) (Table 1).

**Table 1:** Comparison of blood biochemical results between the two groups.

	PTH (ng/L)	Calcium (mmol/L)	Phosphorous (mmol/L)
Adenoma group (n = 19)	379.15 ± 78.63	1.61 ± 0.49	2.25 ± 0.17
Hyperplasia group (n = 38)	186.57 ± 87.00	1.38 ± 0.46	2.17 ± 0.15
t	7.005	1.73	1.709
*P* value	<.001	.089	.093

PTH = parathyroid hormone.

### 3.2. Comparison of lesion location, volume, and blood flow grades between the groups

The volume and blood flow grades significantly differed between the adenoma and hyperplasia groups (*P* < .001); however, the location distribution did not significantly differ (*P* > .05) (Table 2).

**Table 2:** Comparison of routine ultrasound between the two groups.

	Adenoma group (n = 19)	Hyperplasia group (n = 38)	t or *χ*2	*P* value
Volume (cm^3^)	0.67 ± 0.20	0.28 ± 0.08	8.088	<.001
Blood flow (n)			16.625	<.001
level < 2	5	31		
level ≥ 2	14	7		
Location (n)			3.219	.359
Left superior lobe	0	4		
Left inferior lobe	6	13		
Right superior lobe	2	6		
Right inferior lobe	11	15		

a.

b.

### 3.3. Comparison of lesion elastic modulus between the 2 groups

The E-min of the adenoma group was 14.62 ± 6.79 kPa, E-mean was 19.42 ± 6.29 kPa, and E-max was 24.25 ± 6.35 kPa which were significantly different from those in the hyperplasia group (*P* < .05, Table 3).

**Table 3:** E-min, E-mean, and E-max results of the two groups of lesions.

	E-min	E-mean	E-max
Adenoma group	14.62 ± 6.79	19.42 ± 6.29	24.25 ± 6.35
Hyperplasia group	11.29 ± 5.32	14.61 ± 5.69	17.37 ± 5.92
t	2.03	2.904	4.04
*P* value	<.05	<.05	<.001

E-min = minimum, E-max = maximum.

### 3.4. Receiver operating characteristic curve analysis

**Figure 3.**
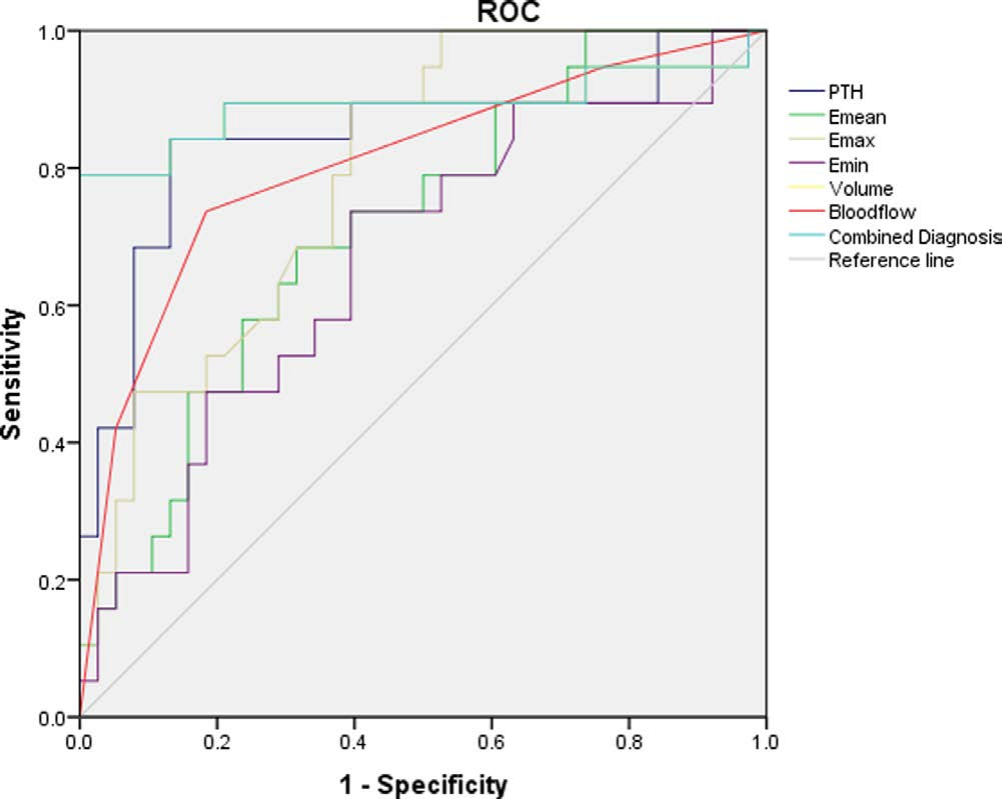
ROC curve of lesion volume, blood flow grade, shear wave elastic modulus, PTH concentrations, and combined diagnosis of parathyroid adenoma. PTH = parathyroid hormone, ROC = receiver operating characteristic.

The optimal cutoff values for diagnosing parathyroid adenoma based on lesion volume, blood flow distribution, PTH concentration, E-min, E-mean, and E-max were 0.475 cm³, level 1.5, 281.85 ng/L, 11.620 kPa, 16.125 kPa, and 17.995 kPa, respectively. The corresponding AUC values were 0.892, 0.805, 0.845, 0.664, 0.715, and 0.792, respectively (*P *< .05). The combinations of more than 6 indicators in the diagnosis of parathyroid adenoma resulted in an AUC of 0.892 (*P* < .001), and the sensitivity and specificity were 78.9% and 97.4%, respectively (Fig. 3).

## 4. Discussion

sHPT results from chronic parathyroid stimulation. It is caused by hypocalcemia, hyperphosphoremia, or vitamin D deficiency, and leads to increased PTH synthesis and the proliferation of parathyroid cells. It is a major complication in patients with end-stage renal failure, especially in those with chronic renal failure requiring hemodialysis.^[7,8]^

The kidney disease outcomes quality initiative guidelines recommend parathyroidectomy in dialysis patients with severe sHPT, especially before kidney transplantation, to prevent cardiovascular complications and ectopic calcification.^[7]^ Patients with sHPT who require surgical treatment range from those with prophylactic parathyroidectomy to those with severe sHPT who do not respond to medication. The surgical methods for parathyroid adenoma and hyperplasia are different. The former mainly involves subtotal resection or total parathyroid resection + autologous transplantation combined with double neck exploration, whereas the latter mainly involves single-gland resection.^[9]^ Therefore, more accurate preoperative positioning and a precise qualitative diagnosis are required to select surgical methods to reduce or avoid postoperative complications.

High-frequency ultrasonography can help visualize lesion morphology and internal echo and dynamically observe the border between the lesion and surrounding tissues and organs in real time. The sensitivity and specificity of this method for locating enlarged parathyroid glands are 69% to 90% and 90% to 98%, respectively.^[10]^ However, when parathyroid adenoma and parathyroid hyperplasia are present on small round or oval ultrasonography with low echo, clear boundary, and prominent blood flow signal, they are difficult to differentiate and diagnose using traditional ultrasonography. Therefore, the development of an objective evaluation method holds considerable clinical significance.

Real time SWE can quantitatively measure the elastic modulus of biological tissues. It is a relatively mature elastic imaging application technology and an important supplement to conventional ultrasound. Real time SWE uses ultrafast ultrasonic imaging technology to track and capture the displacement of each point on the shear wave propagation path in real time, obtain the elastic modulus value of the parathyroid gland through a quantitative analysis system, and obtain real time tissue elastic imaging images through color-coding technology.^[11]^ The higher the value, the faster the shear wave velocity and the greater the hardness of the tissue. The shear wave propagation velocity varies in different biological tissues owing to their different molecular structures; therefore, the elastic degree of different biological tissues can be evaluated by measuring the elastic modulus value.^[11]^ In this study, we observed no significant differences in blood calcium or phosphorus levels between the hyperplasia and adenoma groups. The blood PTH concentration in the adenoma group was significantly higher than that in the hyperplasia group. The lesion volume and blood flow grade were also significantly higher in the adenoma group than in the hyperplasia group. The cutoff values for the blood PTH concentration, lesion volume, and blood flow grade were 281.85 ng/L, 0.475 cm³, and grade 1.5, respectively. This is because during the development of severe hypertrophy and hypertrophy of the parathyroid glands resulting in adenomatous changes, tumor metabolism is active and sufficient nourishment is provided by the new and abundant network of capillaries.^[12]^ Therefore, blood supply to the glands is increased, as demonstrated by color Doppler imaging.

The amount of adipose tissue is higher in the normal parathyroid stroma, and parathyroid hyperplasia is dominated by mixed hyperplasia of the chief cells, eosinophils, and clear cells. Fat can be seen in the stroma, usually without a fibrous capsule, whereas parathyroid adenoma is dominated by chief cells, tightly arranged, with reduced fat content in the stroma, and a fibrous septum with a complete fibrous capsule.^[13]^ The progression from hyperplasia to adenoma significantly alters the internal composition and structure of the parathyroid gland, with significant decreases in adipose tissue and significant increases in fibrous tissue and tissue hardness. Our findings suggested that the values of E-max, E-mean, and E-min in the adenoma group were significantly higher than those in the hyperplasia group, indicating that the hardness of parathyroid adenoma was greater than that of parathyroid hyperplasia, which is consistent with the pathological changes.^[14,15]^

The results indicated that the volume, blood flow, PTH concentration, and elastic modulus of the joint focus were of high value in diagnosing parathyroid adenoma. The AUC was 0.892 (*P* < .001), sensitivity was 78.9%, specificity was 97.4%, and ultrasonic elastic imaging was a simple, operator-independent, repeatable method. Thus, ultrasonic elastic imaging can be used to distinguish adenomas from parathyroid gland hyperplasia.

This study had certain limitations. First, the number of patients was small, and the results may have been biased. However, pathological results obtained for all enrolled patients confirmed the presence of parathyroid lesions. Second, ultrasound elasticity was limited to some extent by tissue depth. In addition, some of the lesions were adjacent to the common carotid artery, which is affected by respiratory and pulse fluctuations. Thus, precise inferences could not be drawn for these lesions. Third, the elasticity values of normal parathyroid glands could not be obtained for the cases in this study, and thus, our findings could not be compared with the parathyroid glands of normal patients.

Nonetheless, the application of ultrasonic elastic imaging to diagnose sHPT has clinical significance. It is a simple, noninvasive, operator-independent, and repeatable method that can be used as a supplement to 2-dimensional and Doppler ultrasound in clinical practice to distinguish parathyroid adenoma from parathyroid hyperplasia. Ultrasonic elastic imaging can provide relevant information on parathyroid elasticity and is a valuable tool for locating parathyroid tumors and hyperplasia in patients with sHPT.

## Author contributions

**Conceptualization:** Xiangyi Li, Yongkang Liu.

**Data curation:** Xiangyi Li.

**Formal analysis:** Xiangyi Li.

**Funding acquisition:** Xiangyi Li, Haowen Li.

**Investigation:** Xiangyi Li, Haowen Li.

**Methodology:** Haowen Li, Yongkang Liu.

**Project administration:** Haowen Li, Yongkang Liu.

**Resources:** Haowen Li.

**Software:** Haowen Li, Yongkang Liu.

**Supervision:** Xiangyi Li, Haowen Li, Yongkang Liu.

**Validation:** Xiangyi Li, Haowen Li.

**Visualization:** Xiangyi Li, Yongkang Liu.

**Writing – original draft:** Xiangyi Li.

**Writing – review & editing:** Xiangyi Li.
